# Prehospital Lactate Measurement by Emergency Medical Services in Patients Meeting Sepsis Criteria

**DOI:** 10.5811/westjem.2016.6.30233

**Published:** 2016-07-21

**Authors:** Lori L. Boland, Jonathan S. Hokanson, Karl M. Fernstrom, Tyler G. Kinzy, Charles J. Lick, Paul A. Satterlee, Brian K. LaCroix

**Affiliations:** * Allina Health, Division of Applied Research, Minneapolis, Minnesota; † Allina Health, Emergency Medical Services, St. Paul, Minnesota; ‡ Abbott Northwestern Hospital, Department of Emergency Medicine, Minneapolis, Minnesota

## Abstract

**Introduction:**

We aimed to pilot test the delivery of sepsis education to emergency medical services (EMS) providers and the feasibility of equipping them with temporal artery thermometers (TATs) and handheld lactate meters to aid in the prehospital recognition of sepsis.

**Methods:**

This study used a convenience sample of prehospital patients meeting established criteria for sepsis. Paramedics received education on systemic inflammatory response syndrome (SIRS) criteria, were trained in the use of TATs and hand-held lactate meters, and enrolled patients who had a recent history of infection, met ≥ 2 SIRS criteria, and were being transported to a participating hospital. Blood lactate was measured by paramedics in the prehospital setting and again in the emergency department (ED) via usual care. Paramedics entered data using an online database accessible at the point of care.

**Results:**

Prehospital lactate values obtained by paramedics ranged from 0.8 to 9.8 mmol/L, and an elevated lactate (i.e. ≥ 4.0) was documented in 13 of 112 enrolled patients (12%). The unadjusted correlation of prehospital and ED lactate values was 0.57 (p< 0.001). The median interval between paramedic assessment of blood lactate and the electronic posting of the ED-measured lactate value in the hospital record was 111 minutes. Overall, 91 patients (81%) were hospitalized after ED evaluation, 27 (24%) were ultimately diagnosed with sepsis, and 3 (3%) died during hospitalization. Subjects with elevated prehospital lactate were somewhat more likely to have been admitted to the intensive care unit (23% vs 15%) and to have been diagnosed with sepsis (38% vs 22%) than those with normal lactate levels, but these differences were not statistically significant.

**Conclusion:**

In this pilot, EMS use of a combination of objective SIRS criteria, subjective assessment of infection, and blood lactate measurements did not achieve a level of diagnostic accuracy for sepsis that would warrant hospital prenotification and committed resources at a receiving hospital based on EMS assessment alone. Nevertheless, this work provides an early model for increasing EMS awareness and the implementation of novel devices that may enhance the prehospital assessment for sepsis. Additional translational research studies with larger numbers of patients and more robust methods are needed.

## INTRODUCTION

Severe sepsis constitutes a major public health burden in the United States, with an estimated 750,000 individuals diagnosed annually, a mortality rate that approaches 30%, and associated annual healthcare costs of $17 billion.^1^ Accelerated time to recognition and definitive treatment has been shown to significantly improve outcomes, and accordingly, a national campaign to develop, endorse, and implement corresponding practice guidelines appears to have reduced mortality among patients with sepsis in hospital settings across the country.^2^, ^3^, ^4^,^5^ But recent reports highlight that 40%–60% of patients presenting to emergency departments (ED) with severe sepsis arrive via emergency medical services (EMS) transport, and that the volume of EMS encounters involving septic patients appears to be outpacing those for myocardial infarction (MI) and stroke.^6^–^9^ EMS recognition and prehospital notification have proven successful in reducing adverse outcomes related to MI and stroke, and thus an analogous case has been made for augmenting the role of EMS in the early detection and care of patients with severe sepsis.^10^–^15^

Commentary about the promising role for EMS in sepsis detection has focused primarily on improving provider education and expanding prehospital diagnostics, with much recent attention given to point-of-care blood lactate testing.^10^, ^14^, ^16^–^18^ The value of blood lactate as a risk stratification tool in sepsis has been demonstrated in ED settings, but the theorized advantage of moving this component of sepsis prediction upstream in the continuum of care remains largely untested.^17^, ^19^–^23^ A handful of studies has established the feasibility of portable lactate meter use in ED triage and prehospital setting, but only one has specifically addressed the use of such devices by prehospital providers to aid in the recognition and early treatment of adult patients with sepsis.^16^,^17^,^24^–^28^ There is a clear need for reports detailing EMS protocols for sepsis, demonstrations of their translation into practice and their association with patient outcomes.

The purpose of this pilot study was to acquire preliminary data and applied-setting experience that would inform the development and formal test of an EMS intervention to improve sepsis recognition through provider education and adjunct diagnostic tools. The specific objectives were the following: (1) to develop and deliver sepsis education for prehospital clinicians, with emphasis on recognition of systemic inflammatory response syndrome (SIRS) criteria; (2) to procure and place hand-held lactate meters and temporal artery thermometers (TATs) in our ambulances and establish protocols and training for their use; (3) to quantify how much sooner an initial blood lactate value could be available to providers in our health system if obtained in the prehospital setting; and (4) to examine, in patients meeting criteria for sepsis, the association between observing an elevated blood lactate (i.e. ≥ 4.0 mmol/L) in the prehospital setting and three specific outcomes: admission to an intensive care unit (ICU), hospital diagnosis of sepsis, and inhospital mortality.

## METHODS

### Setting

This prospective pilot study was a collaborative effort between an ambulance service and two hospital EDs belonging to a single health system in greater Minneapolis. The ambulance service provides 911 dispatch service, advanced life support, basic life support, and scheduled medical transport in 100 communities in and around Minneapolis-St. Paul, Minnesota. The agency employs 430 emergency medical technicians (EMTs) and paramedics and responds to approximately 90,000 calls annually across a 1,200 square mile coverage area. An electronic prehospital patient care record (ePCR; Imagetrend^™^) was fully implemented in early 2008. The two participating EDs – one located in a 639-bed tertiary hospital in Minneapolis and the second in an 86-bed hospital in a suburb of the Shakopee – handle a combined 75,000 emergency visits and approximately 500 patients who meet criteria for severe sepsis or septic shock annually.

Study funding did not allow for full-scale implementation of the project across our ambulance service, so a reduced-scale approach was devised. TATs and lactate meters were available only on ambulances used in the portion of the service area most likely to transport patients to the participating EDs, and only a subset of the system’s paramedics/EMTs (hereafter, paramedics) received sepsis education and protocol training, with priority given to paramedics who primarily practiced in the defined study coverage area.

### Devices

The hand-held whole blood lactate analyzer selected for use was the LactatePro (KDK Corp, Kyoto, Japan) which has been described previously.^24^ Briefly, this FDA-approved, battery-powered device uses disposable test strips and produces a whole blood lactate value in 60 seconds. In this prehospital application, per device specifications, a capillary blood sample from a fingerstick was used for analysis. An FDA-approved temporal artery thermometer (TAT; TAT-5000, Exergen Corp., Watertown, MA) was used by paramedics to assess body temperature non-invasively. Use of this temporal artery scanner involves gently sliding the device across the forehead and then momentarily placing it on the neck area behind the ear lobe. Results are produced in less than five seconds. Ten study kits, each containing a TAT, a lactate meter, and a supply of lactate test strips, were assembled and placed on ambulances in the participating portion of the ambulance service area.

### Provider Training

In June 2011, 37 paramedics attended a two-hour training session during which they received information about the scope of sepsis in the U.S., education about the risk factors and early signs and symptoms of sepsis, and a review of the objectives and protocol for the study. Attendees were also instructed on use of the study data collection tool and proper use of the devices, including a practical component. Hangtags displaying the study eligibility criteria and key aspects of the protocol were distributed. Six months after the start of the pilot, the same clinicians attended a brief study refresher course.

### Study Protocol

Patients meeting the conventional definition of sepsis (i.e. two or more SIRS criteria plus evidence of infection) were targeted for study. Consideration for enrollment was triggered when paramedics encountered a patient with a reported history of recent infection or when a recent infection was suspected. Patients were required to be ≥ 18 years of age, not pregnant, and destined for transport to one of the two participating hospitals. In patients meeting these initial criteria, paramedics then measured body temperature and ascertained heart rate and respiratory rate to further assess eligibility. Patients were enrolled if two or more of the following SIRS criteria were confirmed: (1) heart rate ≥ 90 beats per minute, (2) respiratory rate > 20 per min, or (3) body temperature < 36° C or > 38° C by TAT. In eligible patients, a prehospital lactate value was then determined using the lactate meter. Per device guidelines, the first drop of blood was discarded and the second drop used for analysis. The paramedics otherwise delivered standard care. Upon hospital arrival, ED staff was notified of the patient’s enrollment in the study and an inhospital lactate test was encouraged but ordered at the physician’s discretion. In both receiving hospital laboratories, enzymatic methods are used to determine the blood lactate value in venous blood that has been spun, separated, and refrigerated within 15 minutes. Paramedics were not instructed to report the prehospital lactate value to the ED physician, but were also not specifically prohibited from doing so. The institution’s internal institutional review board approved the study protocol with a waiver of patient consent.

### Data Collection and Definitions

Paramedics recorded study-specific prehospital data in a secure online study database accessible at the point of care. The study database was then linked with data from the hospital electronic health record (EHR) to obtain information on the following: ED lactate collection time and value, inpatient admission subsequent to ED care, sepsis diagnoses, length of stay, and mortality.

Prehospital clinicians categorized the type of infection and indicated the source of their knowledge or suspicion of infection as patient self-report, bystander report, or EMS observation only. An elevated lactate was defined as ≥ 4.0 mmol/L. Hospital admission was defined as admission to an inpatient unit after the ED encounter. Formal diagnoses of sepsis were determined by reviewing the International Classification of Diseases 9^th^ Revision (ICD-9) hospital discharge codes, and included the following: septicemia (any 038), sepsis (995.91), severe sepsis (995.92), and septic shock (785.52). Time stamps were used to compute the time interval in minutes between the prehospital measurement of lactate and (1) patient arrival in the ED, (2) blood specimen collection in the ED, and (3) time the ED lactate value was posted in the hospital EHR.

### Analysis

We described patient characteristics and prehospital findings using means and proportions. Hospital admission, sepsis diagnosis, mortality and mean length of stay were examined overall and by the presence or absence of an elevated prehospital lactate value (i.e. ≥ 4 mmol/L), with Fisher’s exact test or a t-test used to evaluate differences by prehospital lactate group for the categorical variables and continuous variable, respectively. We computed unadjusted Pearson correlation coefficients to explore agreement between prehospital and ED values of lactate and body temperature. Time intervals were described using medians.

## RESULTS

**Figure 1:**
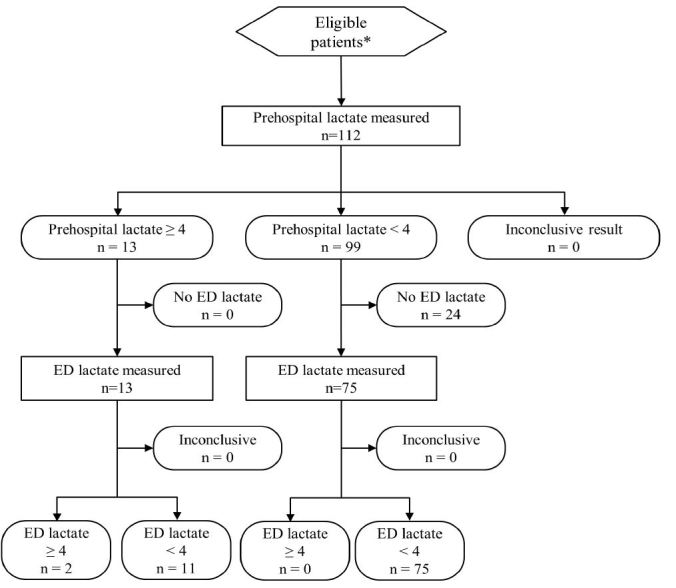
Flow diagram of patient prehospital and emergency department (ED) lactate measurements.

A total of 112 patients were enrolled between July 2011 and August 2013 (Figure 1). Prehospital lactate values ranged from 0.8 to 9.8 mmol/L with an elevated prehospital lactate documented in 13 patients (12%; Table 1). Only two of the 13 patients with prehospital lactate ≥ 4.0 mmol/L had lactate levels that remained ≥ 4.0 mmol/L when assessed by ED staff. Ambulance transport times ranged from 3 to 37 minutes with a mean of 19 minutes. One-third of the patients received intravenous fluids as part of prehospital care (Table 1), with only four patients receiving > 250cc normal saline. Half of the study patients received supplemental oxygen as part of prehospital care in order to achieve and maintain oxygen saturation levels > 94% per standard protocol.

**Figure 2:**
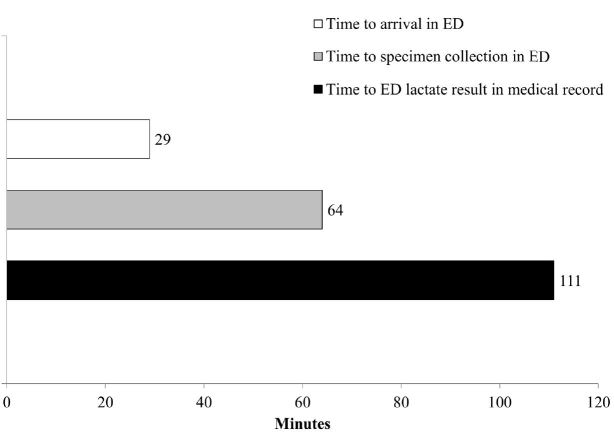
Median time interval (minutes) between prehospital lactate measurement and acquisition of emergency department (ED) lactate value.

Among the 88 patients who had lactate measured in the ED, the median (25^th^, 75^th^ percentile) interval of time that elapsed between the prehospital lactate measurement and ED specimen collection was 64 minutes (50, 84; Figure 2). Within our system, the median interval of time between the paramedic assessment of blood lactate and the electronic posting of the ED-measured lactate value in the hospital EHR was 111 minutes (Figure 2).

Overall, 81% of study patients were hospitalized, 24% received a diagnosis of sepsis, and 3% died during hospitalization (Table 2). Subjects with prehospital lactate ≥ 4.0 were somewhat more likely to have been admitted to the ICU, to have been diagnosed with sepsis, and to have died during hospitalization than those with lactate levels < 4.0, but these differences were generally not statistically significant. Only 5 of the 27 patients who ultimately received a diagnosis of sepsis had a prehopsital lactate ≥ 4.0 mmol/L (19% sensitivity), while 77 of the 85 patients who did not receive a diagnosis of sepsis had a prehospital lactate < 4.0 mmol/L (91% specificity). Results from a sensitivity analysis using a prehospital lactate cut point of 2.5 mmol/L were similar (Table 2). Detailed data on the 13 subjects with prehospital lactate values ≥ 4.0 reveal that the two patients who ultimately died during the index hospitalization were among the oldest subjects, and had comparably low prehospital oxygen saturation levels (Table 3).

Although not systematically studied, we offer several qualitative comments regarding implementation of the selected devices. At the time the study was conducted, a lactate meter, 20–30 lactate test strips, and a TAT cost about U.S. $900. Study paramedics made no reports of malfunction or difficulties in using the LactatePro, but the device has since been discontinued. As it is currently the only hand-held lactate analyzer approved for medical use in the U.S., this presents a significant barrier for EMS systems seeking to introduce lactate assessment. There were three cases where paramedics noted that the TAT would produce only an error message, one occurring in an extremely cold ambient temperature, a phenomenon that has been reported previously.^29^

## DISCUSSION

A compelling case has been made for increasing EMS involvement in the recognition and care of patients with severe sepsis and septic shock, but we have identified only one report that has detailed the implementation of and preliminary experience with a specific sepsis-targeted EMS educational curriculum and treatment protocol.^10^–^15^,^25^ The practical execution of EMS sepsis programs, particularly those that incorporate diagnostic tools not traditionally available in the prehospital setting, should be an area of focus for contemporary EMS research agendas. We educated a small set of EMS providers about sepsis and SIRS criteria, and equipped them with devices to measure body temperature and lactate level to facilitate a more complete prehospital assessment for sepsis.

This preliminary report describes outcomes in a small convenience sample of patients treated and transported by EMS who met established criteria for sepsis, a subset of whom had prehospital whole blood lactate values ≥ 4.0 mmol/L. As this cut point provides the definition of septic shock, one of our goals was to gain some early understanding of what EMS observation of this level of elevated lactate might warrant in terms of pre-arrival communications and/or augmented prehospital care.^30^ We observed that only 2 of the 13 patients with prehospital lactate ≥ 4.0 mmol/L had lactate levels that remained ≥ 4.0 mmol/L when assessed by ED staff, and that 22% of patients with prehospital lactate < 4.0 mmol/L received a diagnosis of sepsis during subsequent hospitalization. Despite demonstration that values obtained by the LactatePro correlate highly with traditional lab-based enzymatic measures, growing concern about the use of capillary samples with hand-held lactate meters has led to an evolution in practice towards the preferred use of venous samples since the time of our study.^31^–^32^ Furthermore, the limitations of single lactate values have been described, and it is clear that not all patients with sepsis mount a lactate response.^20^,^33^,^34^ Seymour et al. documented that among 216 patients transported by EMS and diagnosed with severe sepsis in the ED, only 50% had ED lactate values ≥ 3.0 mmol/L.^34^ Based on our experience, the notion of paramedics using a specified cut point of a single prehospital lactate value as an objective, singular trigger for pre-arrival alert processes, even in the presence of SIRS criteria, cannot yet be supported.

While not a panacea for definitively establishing the population of patients with sepsis in the prehospital setting, EMS evaluation of blood lactate in a variety of patients still has merit for three reasons: (1) it yields a marked reduction in time to a known lactate value; (2) it establishes an earlier initial value for serial assessment; and (3) it can assist in the identification of patients with cryptic shock (i.e. normotension concurrent with tissue level hypoperfusion). In our system, for example, acquisition of a prehospital lactate value would enable an emergency physician to recognize clearance or accumulation of lactate over the previous hour, rather than being limited to a single measurement taken at or near the time of ED arrival. And lactate change, to the extent that it reflects correction or exacerbation of tissue hypoxia, may have more diagnostic relevance than single values. In a Dutch study where blood lactate values were measured an average of 27 minutes apart during the prehospital phase of care, a decrease in lactate between the two time points was associated with decreased mortality.^16^ Nguyen et al. also found that lactate clearance in the first six hours after ED arrival was inversely associated with mortality.^20^ The change in blood lactate between the prehospital and ED measure ranged from −7.9 mmol/L to +1.4 mmol/L in our patients, but given important differences in the methods used in each setting (e.g. the use of capillary versus venous blood), and prehospital interventions during transport, we were unable to interpret lactate change in our study with confidence. Nevertheless, we have demonstrated that the acquisition of early blood lactate for purposes of serial assessment can be extended into the prehospital setting.

While 81% of study patients were hospitalized after the EMS encounter, only a quarter of the study patients received a diagnosis of sepsis during their hospitalization despite being enrolled based upon conventional criteria for sepsis. Indeed, the sensitivity and construct validity of using ≥ 2 SIRS criteria in defining severe sepsis has recently been challenged, but there are several study factors that also may have influenced this result.^35^ First, we made no attempt to validate the presence or suspicion of infection in study subjects. The source of information about infection was noted by paramedics as “EMS observation only” in nearly half of enrolled patients, so inaccuracies in provider perceptions may have resulted in the inclusion of study subjects who had no exposure to recent infection. Second, our definition of diagnosed sepsis included only cases with explicit diagnosis codes (i.e. 038, 995.91, 995.92, or 785.52), and did not include cases of implicit sepsis inferred by the combination of infection with organ dysfunction, as described by Filbin et al.^36^ Third, inclusion in the study was driven strictly by the patient meeting defined, objective criteria, and did not take into account the paramedic’s impression of acuity of illness. Pursuant to the common expression in sepsis “I’ll know it when I see it,” some latitude for clinical acumen and subjective impression may need to be incorporated into EMS sepsis recognition strategies to improve sensitivity, but this requires further study.

## LIMITATIONS

As a pilot study, this work was subject to a number of limitations. There was no attempt to evaluate, measure, and document the acquisition of knowledge and skills related to provider education and training. Providing education and devices to only a small subset of our paramedics limited our capacity for patient enrollment. There was no systematic surveillance of EMS encounters to ascertain whether patients eligible for enrollment were missed by paramedics. There were very few patients with abnormal prehospital lactate values, which limited our ability to examine the relevance of elevated lactate, and the use of capillary versus venous blood samples in the prehospital and ED lactate measurements respectively, prevented rigorous assessment of lactate change. Hospital discharge codes were used to determine a diagnosis of sepsis without consideration for date of diagnosis, so some patients may have developed sepsis later in their hospital stay. Finally, we did not test a specific prehospital care intervention, as the objective was simply to compile early observations of protocol execution and device feasibility in our EMS system.

## CONCLUSION

We attempted to install a test process in our ambulance service whereby EMS could augment the ED care of the septic patient through early recognition. The EMS protocol we piloted, which encompassed objective SIRS criteria, subjective assessment of infection, and body temperature and blood lactate measurements, did not achieve a level of diagnostic accuracy in identifying patients with severe sepsis or septic shock that would warrant pre-arrival alert and committed resources and response at the receiving hospital based on EMS assessment alone. This work provides a preliminary model for increasing EMS awareness of sepsis and implementing novel devices that can add to the completeness of an EMS assessment for sepsis, but additional translational research studies that include larger numbers of patients and more robust methods will be essential in shaping protocols for reliable, early detection of sepsis by EMS clinicians.

## Figures and Tables

**Table 1:** Patient characteristics and prehospital findings.

Variable	Patients (n=112)
Age, y	74 (22 – 100)
Age ≥ 75	58% (65)
Male	45% (50)
History of infection ascertained via	
Patient self-report	29% (32)
Bystander report	24% (27)
EMS observation only	47% (53)
Type of infection	
Respiratory	36% (40)
Gastrointestinal/abdominal	8% (9)
Skin/wound	15% (17)
Urinary tract	19% (21)
Unknown	22% (25)
SIRS Criteria	
Heart rate ≥ 90 bpm	78% (87)
Respiratory rate > 20 pm	77% (86)
Body temperature	
< 36°C or > 38°C	54% (61)
< 36°C	8% (9)
> 38°C	46% (52)
Prehospital blood lactate (mmol/L)	
Mean (SD)	2.6 (1.7)
Median	2.1
Range	0.8 – 9.8
Patients with value ≥ 4.0 mmol/L	12% (13)
Received prehospital IV fluids	33% (37)

*SIRS,* systemic inflammatory response syndrome; *SD*, standard deviation; *IV,* intravenous

* Results are expressed as mean (range) or percent (n) unless otherwise indicated.

**Table 2:** Primary outcomes, overall and by prehospital lactate value.

		Prehospital lactate ≥ 4.0 mmol/L			Prehospital lactate ≥ 2.5 mmol/L		
			
Variable	All (n = 112)	No (n = 99)	Yes (n = 13)	*p Value*^a^	No (n = 69)	Yes (n = 43)	*p Value*^a^
Hospital admission	81% (91)	81% (80)	85% (11)	0.74	77% (53)	88% (38)	0.14
Admitted to ICU	16% (18)	15% (15)	23% (3)	0.44	14% (10)	19% (8)	0.60
Diagnosis of sepsis	24% (27)	22% (22)	38% (5)	0.30	22% (15)	28% (12)	0.50
Length of stay^b^ (days)	4.8 (3.7)	4.9 (3.8)	4.2 (2.2)	0.55^c^	4.8 (3.4)	4.8 (4.1)	0.95^d^
Death during hospitalization	3% (3)	1% (1)	18% (2)	0.04	2% (1)	5% (2)	0.57

*ICU*, intensive care unit

Results are expressed as mean (SD) or percent (n)

a Fisher’s exact test (categorical variables) or t-test, for difference by prehospital lactate category

b Among those admitted to inpatient hospital unit after emergency department encounter

c Mean difference 0.72 (95% CI: −1.65, 3.08)

d Mean difference −0.05 (95% CI: −1.62, 1.52)

**Table 3:** Lactate values (mmol/L), prehospital findings, and hospital outcomes of enrolled patients with prehospital lactate ≥ 4.0 mmol/L.

			Lactate values		Prehospital findings							Hospital outcomes	
					
Patient	Age	Gender	Prehospital	ED	Infection type	HR	RR	Body temp	Lowest O2 saturation	Lowest SBP	IV fluids	ICD-9 Diagnosis codes for sepsis*	Inhospital death
1	92	F	5.7	2.2	Respiratory	152	40	99.9	77	102	Yes	S1, S3	Yes
2	86	M	9.7	3.3	Respiratory	130	45	101.1	72	92	Yes	S1, S3, S4	Yes
3	54	M	7.0	1.7	GI/abdominal	97	20	98.6	98	150	No	None	No
4	66	M	8.0	5.9	Other	111	34	99.9	98	150	Yes	S1, S2	No
5	22	F	4.6	1.6	Urinary	122	26	97.9	98	146	Yes	None	No
6	83	F	5.1	2.5	GI/abdominal	104	24	-	93	98	No	None	No
7	80	M	5.0	1.7	GI/abdominal	108	28	99.7	94	120	Yes	None	No
8	89	F	9.8	1.9	Other	106	22	97.7	97	128	No	None	No
9	80	M	4.7	1.8	Other	85	-	102.6	86	89	Yes	None	No
10	55	M	4.0	1.9	Respiratory	122	38	102.6	87	120	Yes	None	No
11	71	M	7.1	5.3	Other	120	22	-	90	69	No	None	No
12	80	F	4.6	0.7	Skin/wound	104	22	-	89	148	No	S1, S2	No
13	42	M	5.4	1.2	Skin/wound	116	41	102.0	96	115	No	S1, S2	No

*ED,* emergency department; *HR,* heart rate; *RR,* respiratory rate; *SBP,* systolic blood pressure; *IV,* intravenous; *ICD-9,* International Classification of Diseases 9th Revision; *GI,* gastrointestinal

* S1 = Septicemia (038.xx), S2 = Sepsis (995.91), S3 = Severe Sepsis (995.92), S4 = Septic Shock (785.52)
